# Physiological Chemistry

**Published:** 1897-12

**Authors:** John A. Miller

**Affiliations:** Professor of chemistry, Niagara University


### PHYSIOLOGICAL CHEMISTRY.

Reported by JOHN A. MILLER, Ph D.,
Professor of chemistry, Niagara University.

### OXYGEN TENSION OF ARTERIAL BLOOD.

JOHN S. HALDANE and J. Lorrain Smith (*J. Physiol.*, 1896 ; *J. Chem. Soc.*, 1897). It was proved that the oxygen tension of human arterial blood is 26.2 per cent. of an atmosphere, or 200 mm. of mercury ; as this is higher than the tension of oxygen in alveolar air, diffusion alone will not explain the passage of oxygen from the alveolar air to the blood.

---

### MUSCULAR POWER AND GASEOUS METABOLISM.

Louis Schnyder (*Zeits. Biol.*, 1896 ; *J. Chem. Soc.*, 1897.) The increased discharge of carbon dioxide that occurs during work is lessened by practice. The amount of decomposition of tissue depends on the extent of the exertion rather than of the work done. In normal individuals the involuntary muscles are already in a state " training," and in weakened convalescents these work with abnormal exertion even during so-called rest.

---

### OCCURRENCE OF INOSITE IN THE THYROID GLAND.

R. Tambach (*J. Pharm.*, 1896 ; *J. Chem. Soc.*, 1897). This substance appears to occur in larger quantity, from 0.5 to 0.8 per cent., in the thyroid gland than in any other part of the body.

### CHEMISTRY OF THE THYROID GLAND.

Robert Hutchison (*J. Physiol.*, 1896 ; *J. Chem. Soc.*, 1897). The thyroid contains two proteids, a nucleo-albumin and the colloid matter; the former is present in small amount and is probably derived from the epithelium. The colloid is contained in the acini. It contains a small amount of phosphorus, a considerable proportion of iodine; it yields no reducing substance on treatment with mineral acids and no nuclein bases, and is, therefore, neither a mucin nor a nucleo-proteid. On gastric digestion it is readily split into a proteid and a nonproteid part; both of these, but especially the latter, contain iodine. The nonproteid part contains all the phosphorus of the original substance. The ordinary extractives are fairly abundant, but the colloid is the active physiological constituent of the gland; both parts of it are active, but the nonproteid part is the more active of the two.

### THE SIGNIFICANCE OF CHLORIDES IN ANEMIA.

Waclaw von Moraczewski (*Virchow's Archiv.*, 1896 ; *J. Chem. Soc.*, 1897). During anemia there is a diminution in the excretion of chlorides in the urine ; the excretion increases as the patient gets better. Calcium phosphate behaves like the chlorides. The alkali phosphates and uric acid are increased in amount in the urine in the anemic period ; this increase lessening with convalescence. An addition of calcium phosphate and sodium chloride to iron salts increases their blood-forming action.

### PHLORIDZIN DIABETES.

F. H. Pavy (*Proc. Physiol. Soc.*, 1896 ; *J. Chem. Soc.*, 1897). The statement has been made that in phloridzin diabetes there is no glycohemia. The present communication shows that if fallacies in the collection of blood, in the use of antiseptics and in the method employed for analysis of the blood be avoided, there is a distinct rise in the percentage of sugar in blood as a result of giving the drug.

### THE PROTEIDS OF LEUCEMIC URINE.

R. Kolisch and R. Burian (*Zeits. Klin. Med.*, 29 ; *J. Chem Soc.*, 1897). Albumosuria is not a constant feature in leucemia ; when present it probably originates from decomposition of leucocytes, the increase in alloxuric substances in the urine supporting this view.

---

CHANGES OF THE FAT OF CHYLE IN THE BLOOD.

WILHELM COHNSTEIN and Hugo Michaelis (*Pflüger's Archiv.*, 1896 ; *J. Chem. Soc.*, 1897). The blood has the property of causing the disappearance of the fat of the chyle, introduced in the natural way or by artificial injection. This depends on the presence of oxygen and is associated with the corpuscles of the blood. The red corpuscles contain a substance which has this *lipolytic* function; the fat is changed into a substance insoluble in ether ; this substance is not gaseous, but solid ; water and carbon dioxide were not found in experiments *in vitro*. The change the fat undergoes is probably saponification ; but further work is in hand on the question.

---

EFFECT OF A MEAL ON THE NITROGEN OF THE URINE.

OTTO VERAGUTT (*J. Physiol.*, 1897 ; *J. Chem. Soc.*, 1897). After a meal rich in proteids, the output of nitrogen in the urine shows three rises, one immediately, the second two to four hours and the third six to seven hours later. If the food is poor in proteid, the three rises are still seen, but are not so well defined. The first rise is the most constant.

---

MULTIPLE INTESTINAL CONCRETIONS IN MAN.

C. T. MÖRNER (*Zeits. Physiol. Chem.*, 1897 ; *J. Chem. Soc.* 1897). The concretions removed from the intestine of a man gave the following results on analysis :

| | |
|---|---|
| Magnesium ammonium phosphate.... | 82.23 per cent. |
| Calcium phosphate.................. | 5.24 " " |
| Magnesium phosphate.............. | 1.64 " " |
| Calcium carbonate.................. | 1.61 " " |
| Calcium soap........................ | 0.75 " " |
| Neutral fat.......................... | 0.20 " " |
| Insoluble organic substance.... ..... | 1.90 " " |
| Water, traces of soluble organic substances, etc...................... | 6.43 " " |

---

CHLORIDES AND PHOSPHATES OF THE BLOOD IN DISEASE.

W. VON MORACZEWSKI (*Virchow's Archiv.*, 1896 ; *J. Chem. Soc.*, 1896). The blood was investigated in a number of diseased conditions of diverse kinds. In pneumonia the chlorides are diminished; they rise after the crisis. The phosphates are abundant; this usual in acute diseases, the urine showing a correspondence. In nephritis there is an increase in the chlorides and a decrease in the phosphates. In lead poisoning, if lead is passing into the urine, the blood is rich in chlorides. The urine is poor in chlorides, rich in phosphates, the opposite to the blood; the same is true for anemia.

---

### PHYSIOLOGICAL ACTION OF CHOLINE, NEURINE AND ALLIED SUBSTANCES.

F. W. Mott and H. D. Halliburton (*Proc. Physiol. Soc.*, 1896; *J. Chem. Soc.*, 1897). When injected into the circulation, small doses of choline hydrochloride cause a marked temporary fall of blood pressure, which is cardiac and not peripheral in origin. It occurs also after section of the vagi. Neurine hydrochloride produces a preliminary fall and a subsequent rise of pressure, respiration being slowed and deepened. This drug is more toxic than choline; respiration ceases before the heart.

The physiological interest of these observations is derived from the fact that the cerebrospinal fluid, in cases of brain disease, where, as in general paralysis of the insane, there is great wasting of the brain substance and disintegration of its cells, produces exactly the same effects as solutions of choline. Normal cerebrospinal fluid is innocuous; the toxicity of the pathological fluid is due to the same nonproteid substance precipitable by phosphotungstic acid. It is probable that this substance choline is derived from the lecithin of the brain. If this is the case, the enfeebled circulation with severe fainting fits and fatty degeneration of the heart, so frequently seen in cases of general paralysis, will be in part accounted for. The blood removed by venesection from patients during the fits contains the same substance.

---

IF GONORRHEA in a female involve the vulva, Horwitz recommends that she be kept perfectly quiet; that the parts be washed with a solution of boracic acid or with a 1,4000 solution of bichloride of mercury, then dusted with an antiseptic powder consisting of calomel and bismuth; and a piece of cotton is to be kept between the labia.—*Am. Jour. Surg. and Gynec.*

